# Obituary: Albert Bendelac (1956-2023), MD, Ph.D

**DOI:** 10.3389/fimmu.2023.1354568

**Published:** 2024-01-12

**Authors:** Kamel Benlagha

**Affiliations:** Université Paris Cité, Institut de Recherche Saint-Louis (IRSL), EMiLy, INSERM U1160, Paris, France

**Keywords:** Albert Bendelac, NK T cells, CD1d, type 1 diabetes, innate T cells

Albert Bendelac, a trailblazer in the exploration of innate T cells and the pioneer who uncovered natural killer (NK) T cells, passed away on 23 August 2023, at the age of 67. Born in 1956 in Casablanca, Albert relocated to France with his family during his teenage years. He earned his MD from University Paris VI in 1985 specializing as a dermatologist.

Albert subsequently joined the laboratory of Jean-François Bach at the Necker Hospital in Paris, where he made groundbreaking contributions to the field of type 1 diabetes. His discoveries included insights into the transmission of the disease by T cells, the CD4 and CD8 dependency of disease progression, and abrogation of disease by MHC class II antibodies. Albert completed his PhD at University Paris VII in 1992 and pursued further training with a postoctoral fellowship in Ron Schwartz’s laboratory at the National Institutes of Health.

In 1995, in collaboration with Olivier Lantz, Albert highlighted a subset of T cells with an invariant T cell receptor, subsequently known as NK T cells, and identified CD1 as their restriction element. During the following decade, while at Princeton University and then at the University of Chicago, he continued to forge valuable collaborations, developing tools such as CD1d teteramers with Luc Teyton and CD1d ligands with Paul B Savage to study NK T cells. Albert also created knock-in and knockout mouse models and T cell hybridomas, contributing to the understanding of CD1d intracellular trafficking and its impact on NKT cell thymic selection, as well as identitying exogenous and endogenous ligands. These seminal discoveries led to his appointment as a Howard Hughes Medical Institute investigator in 2005.

In 2008, Albert identified the transcription factor PLZF as master regulator of NK T cells, mucosal-associated invariant T cells, and later demonstrated its similar role in the develoment of innate lymphoid cells. More recently, he delved into fundamental questions in immunology, exploring the development of intra-epithelial intestinal lymphocytes and the antigen specificity of mucosal IgA produced by an innate B cell population.

Beyond his exceptional research contributions, Albert excelled as a captivating teacher, receiving accolades such as the Quantrell Teaching Award for Excellence in undergraduate teaching at the Univeristy of Chicago in 2019. He was an exemplary mentor, challenging both himself and those he worked with. Albert dedicated himself to training postdocs, establishing tutorial and summer courses on fundamendal questions in immunology. He cared deeply about their future and made positive contributions to their individual careers. He unselfishly shared his ideas, tools, and protocols to both those around him and scientists globally.

Albert, though reserved, possessed a sharp wit. He enjoyed sports, regularly running with his dog, and had a passion for good cuisine, often preparing meals himself. The scientific community, his friends, and his close family, including his wife Bana and their children, Aude, Raphaëlle, and Julien, will dearly miss Albert.


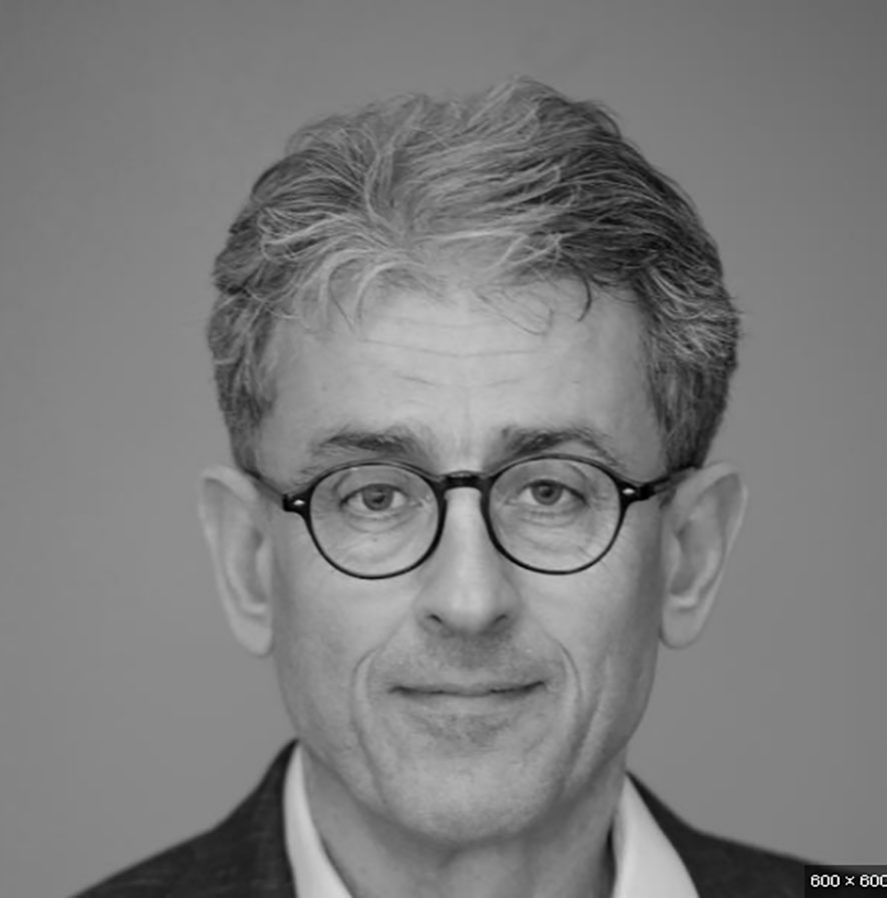


Albert Bendelac. Reproduced with permission from the University of Chicago, available at: https://pathology.uchicago.edu/news/bendelac-quantrell.

## Author contributions

KB: Writing – original draft, Writing – review & editing.

